# Radiofrequency thermal ablation of giant placental chorioangioma complicated with fetal hydrops: a case report and successful outcome

**DOI:** 10.1515/crpm-2023-0028

**Published:** 2024-03-20

**Authors:** Jack Le Vance, Leo Gurney, Shireen Meher, Robert Negrine, Victoria Hodgetts Morton, Tamas Marton, R. Katie Morris

**Affiliations:** Institute of Applied Health Research, 522411 University of Birmingham, Birmingham, UK; Department of Maternal and Fetal Medicine, 1729 Birmingham Women’s and Children’s NHS Foundation Trust, Birmingham, UK; Department of Neonatology, 1729 Birmingham Women’s and Children’s NHS Foundation Trust, Birmingham, UK; Department of Cellular Pathology, 1729 Birmingham Women’s and Children’s NHS Foundation Trust, Birmingham, UK; Department of Obstetrics and Gynecology, Faculty of Medicine, Semmelweis University, Budapest, Hungary

**Keywords:** chorioangioma, hydrops, laser therapy, placental tumour, radiofrequency ablation

## Abstract

**Objectives:**

Chorioangiomas are the most frequently occurring type of benign tumour of the placenta. However, large chorioangiomas greater than 4 cm are rare and can be more frequently associated with serious complications such as: polyhydramnios, hydrops fetalis, fetal anaemia, intrauterine growth restriction, preterm birth, and an increased risk of perinatal mortality. Importantly timely prenatal diagnosis with close surveillance alongside potential intrauterine intervention can prove impactful on pregnancy outcome and fetal survival.

**Case presentation:**

We present a case of a 36-year-old female referred to our tertiary fetal medicine unit at 28 weeks’ gestation with a large chorioangioma measuring 9.4×8.8×5.5 cm and ultrasonographic evidence of severe fetal anaemia and fetal hydrops. The patient underwent an intrauterine transfusion and *in utero* surgical therapy with radiofrequency ablation (RFA). Immediately following the procedure, the fetus sustained a period of bradycardia, followed by asystole. Delivery was expedited via emergency caesarean section. Careful planning and rapid delivery after fetal intervention within the most appropriate surgical setting mitigated risks for the baby and resulted in a positive outcome. The baby was discharged from the neonatal unit on day 84 of life.

**Conclusions:**

Large placental chorioangiomas are a rare occurrence, however, when associated with fetal complications present a high incidence of adverse perinatal outcomes. *In utero* interventions require careful planning and surgical expertise to ensure improved fetal and neonatal outcomes. To the best of our knowledge this case is the first recorded instance of a successful postnatal outcome following RFA for a large placental chorioangioma, whereby the fetus was complicated by fetal hydrops.

## Introduction

Chorioangiomas are the most frequently occurring type of non-trophoblastic benign tumour of the placenta, reported to be detected in 1 % of placentas examined microscopically [1]. Such placental tumours are characteristically small, usually measuring less than 4 cm. Clinical presentation may often be asymptomatic and overlooked in routine ultrasound examination. However, chorioangiomas measuring greater than 4–5 cm, referred to as ‘giant’ tumours, can be associated more frequently with serious complications such as polyhydramnios, hydrops fetalis, fetal anaemia, intrauterine growth restriction, preterm birth, and an increased risk of perinatal mortality [2]. Large placental chorioangiomas are rare within obstetric practice, reported to occur in approximately one in 3,500–9,000 births [3].

Complications are thought to result from chronic placental arterio-venous shunting leading to the development of a high fetal cardiac output, alongside sequestration of erythrocytes and platelets into the placenta [4]. Regular ultrasound assessment with the inclusion of colour flow Doppler imaging plays a vital role in the diagnosis and antenatal monitoring of these tumours. Prenatal ultrasound examination can reveal a well circumscribed mass that is either homogenous or heterogenous in nature [5]. Utilisation of colour flow Doppler can highlight the vascular architecture of the placental tumour and differentiate chorioangiomas from other tumour subtypes [6]. Importantly early prenatal diagnosis, close surveillance and timely intrauterine intervention has the potential to improve pregnancy outcome and fetal survival.

Literature clearly documents the association between placental chorioangiomas and adverse perinatal outcomes [5], [6], [7]. To try to improve outcomes several intrauterine treatments have been attempted and reported in small numbers within the literature, which report varying degrees of operative success [6, 8], [9], [10]. Different approaches include laser ablation, bipolar coagulation, vessel embolization, radiofrequency ablation, surgical ligation and alcohol ablation. As such, there is currently no clear evidence of superiority of any interventions to guide management of placental chorioangiomas. We report a prenatally diagnosed case of a large placental chorioangioma that was referred to our tertiary fetal medicine centre and underwent interventional therapy.

## Case presentation

A 36-year-old female was referred urgently from her local secondary care hospital at 28 weeks’ gestation following the detection of a large placental chorioangioma with associated ultrasound abnormalities suggestive of fetal anaemia (raised middle cerebral artery peak systolic velocity (MCA PSV) of 2.34 multiples of median (MoM)), fetal ascites and polyhydramnios (maximum vertical pool of 11.2 cm). Her blood group was AB Rhesus positive. She previously had three uncomplicated term vaginal births and 2 s trimester losses at 17 and 20 weeks respectively. Earlier within this pregnancy she had received one positive anti-cardiolipin antibody (ACA) IgG result and was commenced on low molecular weight heparin (LMWH) and aspirin to continue throughout.

**Figure 1:**
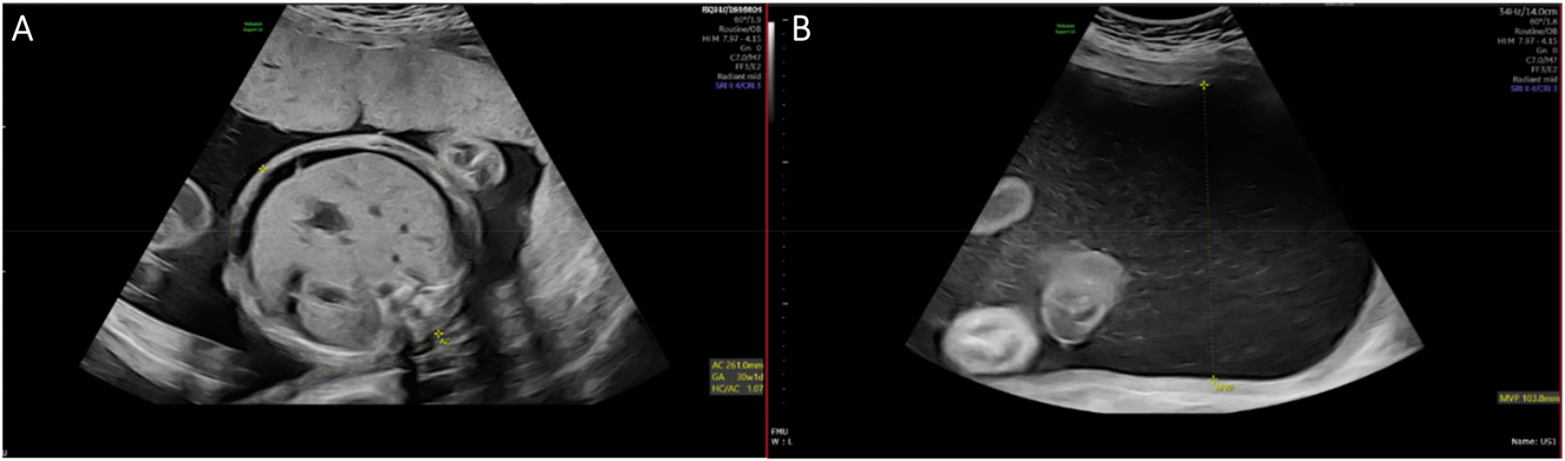
(A) Fetal ascites visualised on ultrasound. (B) Maximal vertical pool depth (MVP) measuring 10.4 cm indicating polyhydramnios.

**Figure 2:**
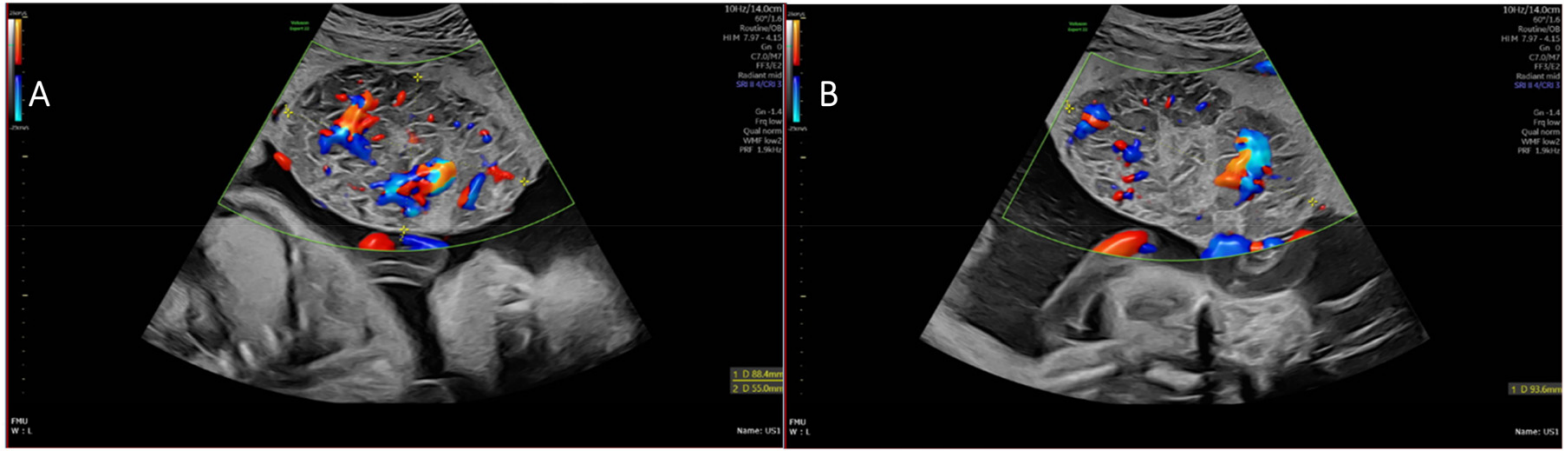
(A and B) Large placental chorioangioma visualised on ultrasound measuring 9.4×8.8×5.5 cm in two dimensions. Colour flow Doppler revealing a large central artery located close to the umbilical cord insertion.

After referral, a detailed ultrasound scan at our tertiary Fetal Medicine Centre at 28 weeks and 5 days gestation demonstrated an appropriately grown male fetus (estimated fetal weight 1,355 g) with marked ascites, skin oedema and polyhydramnios of 10.4 cm (Figure 1A and B). Upon assessment of the anteriorly positioned placenta, a large centrally located, well circumscribed mass measuring 9.4×8.8×5.5 cm was identified (Figure 2A and B). Colour flow Doppler illustrated a large central artery within the tumour, neighbouring the umbilical cord insertion, confirming the suspicion of a large placental chorioangioma. Fetal Doppler waveforms exhibited a raised right MCA PSV 60.84 cm/s (1.6 MoM) indicating severe fetal anaemia. Umbilical artery and ductus venosus waveforms were normal. Assessment of the fetal heart architecture demonstrated marked cardiomegaly with evidence of tricuspid regurgitation, indicating cardiodynamic dysfunction.

Management options were discussed with the patient and partner, which included either urgent delivery and postnatal treatment or intrauterine transfusion (IUT) with the possibility of further *in utero* treatment to ablate the tumour or continued antenatal surveillance. An agreed joint decision was taken to proceed with an IUT and additional intrauterine interventional therapy. Considering the gestational age and MCA PSV, the fetal haemoglobin estimation was 6.1 g/dL, indicating significant fetal anaemia. Calculations determined an IUT of 70 mL would be sufficient at the time of operation [11]. Prenatal steroids were administered prior to the procedure to enhance fetal lung maturity with a view to proceeding with *in utero* treatment the following day.

The modality of intrauterine surgical intervention to ablate the chorioangioma was selected following discussions with two other United Kingdom (UK) fetal medicine interventional centres (Kings College Hospital, London, UK and University College London Hospital, London, UK), that have prior experience of central vessel ablation for chorioangiomas. Consideration was given between using either fetoscopic laser ablation or radiofrequency ablation (RFA). RFA was chosen as the preferred intervention due to the concerns of potential higher risk of central vessel rupture with the use of laser ablation. Additionally, there was a previously published case report using RFA that demonstrated a successful outcome [12].

**Figure 3:**
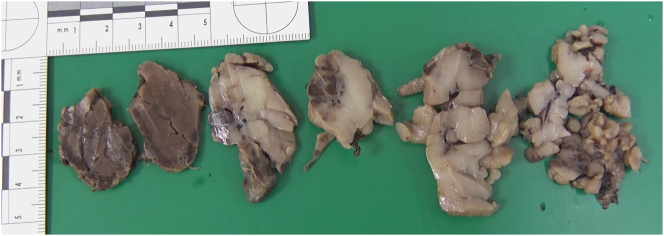
Cross section of the tumour macroscopically. The left two slices show coagulated areas of tumour. The two pieces in the middle shows borderline area between the unaffected and coagulated parts. The two pieces on the right demonstrate the lobulated characteristics of the tumour.

At 28 weeks and 6 days gestation the patient underwent an IUT into the cord root within obstetric theatres, followed by three cycles of RFA. Due to the fetus’ viable gestation and weight, a full obstetric surgical and anaesthetic team were present along with the neonatal team. The IUT occurred successfully, increasing the fetal haemoglobin to 12 g/dL. During each cycle of RFA the vasculature of the placental tumour was thoroughly assessed with colour flow Doppler. Following two cycles of RFA the central tumour artery continued to display active flow, therefore a third cycle of RFA was undertaken. After completion of the third cycle of RFA the fetus sustained a period of bradycardia, with eventual asystole evident on ultrasonography. Consequently, an emergency caesarean section was performed immediately under general anaesthetic, with the baby taken by the neonatal team for resuscitation following prompt umbilical cord clamping. Apgar scores following delivery were 0 at 1 min, 3 at 5 min and 6 at 10 min. Following the third round of intermittent positive pressure ventilation (IPPV), a slight degree of chest movement was visualised, and the fetal heart rate rested between 60 and 80 beats per minute. The baby was intubated by the neonatal team and given Curosurf, whereby oxygen saturations were then maintained between 80 and 90 % on 100 % fraction of inspired oxygen (Fi02). The baby, weighing 1,440 g (g) (68th centile) was transferred to the neonatal intensive care unit (NICU) for further organ support, post-operatively the placenta was sent for histopathological analysis. Macroscopically the placenta was examined fresh. The placenta was ovoid in shape measuring 19×17×2 cm and weighing 322 g. The umbilical cord measured 41×1.5 cm with three vessels on the cut surface and inserted 7 cm from the edge. The membranes were noted to be complete. The fetal surface was normal; however, the maternal surface was incomplete and fragmented. Beneath the umbilical cord insertion, there was an area of missing placental tissue measuring approximately 12×8 cm in diameter. From this area fragmented tumour tissue was identified weighing 191 g, which was in pieces and completely detached from the main placental tissue (Figure 3). The cut surface of the tumour was mostly homogenous and pale red in colour. However, focally the tumour was haemorrhagic and dark brown in colour, as a result of the intervention. The rest of the placenta was spongy, red, with no further focal lesions visible.

**Figure 4:**
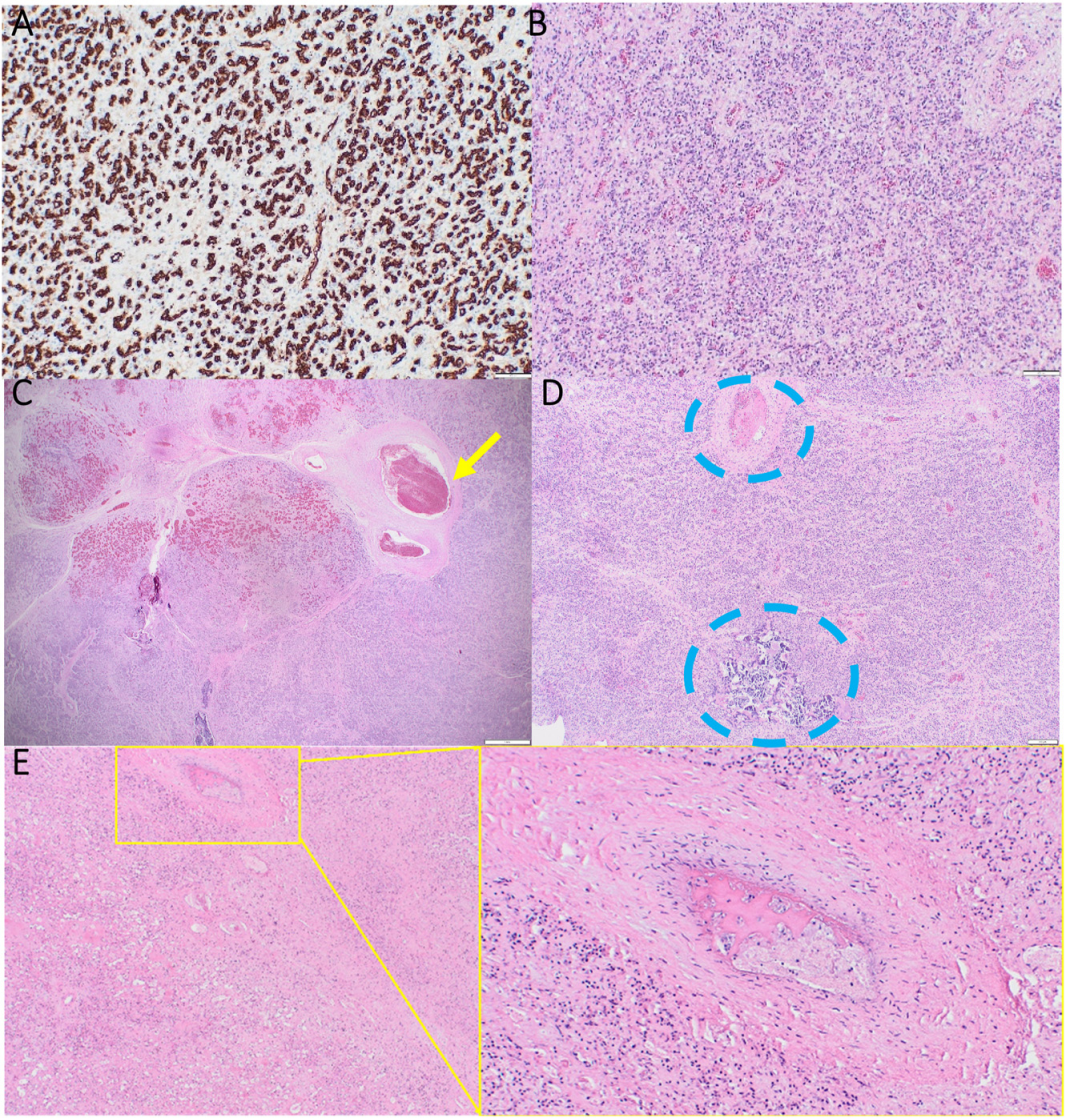
(A) CD31 positive immune staining at 10× magnification, demonstrating multiple capillaries closely packed within the tumour. (B) H&E staining at 10× magnification, demonstrating multiple capillaries closely packed within the tumour. (C) Low magnification image of a tumour lobule with a feeding vessel (arrow). (D) Thrombosed vessel (upper circle) and a small focus of coagulative necrosis in an otherwise unaffected area (lower circle). (E) Coagulation of the tumour with a necrotic area with a thrombosed vessel (enlarged on bottom right).

Microscopically, the umbilical cord, membranes and the unaffected placenta tissue were unremarkable, whereby villous morphology was consistent with the given gestation. The large tumour consisted of CD31 positive capillaries, closely packed in a large lobulated tumour (Figure 4A and B). Within the middle of the tumour lobules, feeding vessels could be clearly visualised (Figure 4C). In some of the feeding vessels fresh fibrin thrombosis was also seen. Pale eosinophilic areas were seen within necrotic areas the interstitium, with reduced nuclear staining, intra lumen fresh thrombus and clear coagulative necrosis (Figure 4D and E). According to the histology approximately 30 % of the total tumour volume was affected by the coagulation.

Postnatally, the neonate required ventilatory support until the fourth day after birth, after which it was transitioned to a continuous positive airway pressure (CPAP) mask for two days and then weaned to high flow oxygen until day 15 of life. An echocardiogram soon after birth revealed a structurally normal heart with indications of elevated pulmonary pressures and myocardial dysfunction. In the immediate postnatal period, multiple ionotropic medications were required to maintain a satisfactory blood pressure, however, successfully tapered off on day three of life. Disseminated intravascular coagulation (DIC) developed soon after birth which corrected following multiple neonatal blood product transfusions and intravenous vitamin K on day one of life. Cranial ultrasounds on day 1 and 2 of life showed no abnormalities, and the retinopathy of prematurity (ROP) screening was normal. However, on day six of life the neonate exhibited multiple aspirates of bilious contents. Additional to this, blood cultures tested positive for *Staphylococcus epidermidis*. A course of intravenous antibiotics was commenced following these results. An abdominal ultrasound displayed moderate ascites, but otherwise normal abdominal viscera. Unfortunately, on day 14 of life worsening abdominal distension and the passage of bloody stools ensued. The condition of the neonate deteriorated, becoming acidotic and hypotensive, which necessitated the requirement for further ionotropic medication. On day 15 of life the neonate was re-intubated and transferred to the local paediatric tertiary unit for potential surgical intervention for suspected necrotising enterocolitis (NEC).

Review of the surgical team deemed that medical management of the suspected NEC would be satisfactory and the neonate was transferred back to our tertiary neonatal unit for ongoing management. On day 20 venous blood and long line cultures grew positive cultures for *Klebsiella*. An escalated course of intravenous antibiotics for neonatal sepsis was commenced following discussion with the microbiology consultant. Congenital thrombocytopenia secondary to neonatal sepsis evolved, requiring multiple platelet transfusions. Reassuringly, an improvement in the haematological and biochemical markers was seen after several days of sepsis treatment. Ventilation occurred until day 27 of life, whereby the neonate was extubated on to bilevel positive airway pressure (BiPAP). On day 28 of life the baby was weaned to CPAP, then high flow oxygen and later, once stable, transferred back to their local hospital on day 40 of life.

A repeat retinopathy screening on day 57 of life demonstrate stage two ROP in both eyes, which requires additional outpatient follow-up. Blood cultures were negative on day 63 of life and antibiotics were subsequently discontinued, with substantial improvement in neonatal condition. The infant was slowly weaned down to 0.2 L per minute of oxygen on day 84 of life and discharge home with follow-up by the home treatment and chronic lung disease teams.

## Discussion

Placental chorioangiomas are the most common type of benign non-trophoblastic tumour of the placenta and embryonically derive from the chorionic mesenchyme [4]. Microscopically, three histological patterns have been documented within the literature: angiomatous, cellular and degenerate [13]. Angiomatous patterns are the most common, typically characterised by multiple minute vascular areas in numerous stages of their development which are encapsulated by placental stroma [8].

Tumour size has been closely related to the development of adverse pregnancy outcomes. A recent meta-analysis of 28 studies (161 pregnancies) revealed the pooled risk for perinatal death, in relation to the size of the chorioangioma measuring ≥2, ≥ 4, ≥ 6, ≥ 8 and ≥ 10 cm was 10.4 % (95 % CI, 3.9–19.5 %) 11.2 % (95 % CI, 4.6–20.3 %),13.9 % (95 % CI, 6.0–24.3 %), 20.6 % (95 % CI, 7.0–39.0 %) 27.9 % (95 % CI, 6.9–56.2 %), respectively [5]. In our case, the tumour size measured 9.4 cm at its widest, indicating a very high risk of fetal mortality based on size alone. As such, early prenatal diagnosis, regular gestational surveillance, and prompt detection of fetal complications are all vital for timely intervention.

Grey-scale ultrasonography is the primary diagnostic modality to assist with early detection and continuation of fetal monitoring. The appearance of a well circumscribed mass that is hyper or hypoechoic in nature, situated closely to the umbilical cord insertion, and which protrudes into the amniotic cavity are typical features of large chorioangiomas [4]. This can clearly be seen within our case, making the diagnosis of a chorioangioma prenatally relatively straight forward. The use of colour flow Doppler can aid in the detection of vascular channels within the tumour that can help clinicians differentiate these tumours from other pathologies such as placental haematomas, teratomas and degenerating fibroids [14], [15], [16].

Smaller chorioangiomas typically present without any fetal or maternal compromise and are usually associated with a good perinatal outcome [1, 5]. Whereas large tumours (>4 cm) have been more frequently associated with significant fetal and maternal complications, as demonstrated within the literature [9, 17], [18], [19], [20]. In accordance with the this, our case history demonstrated ultrasonographical findings of mild polyhydramnios, marked cardiology and features of fetal hydrops.

Chronic arteriovenous shunting and an increased fetoplacental blood volume, as a result of the chorioangioma, can lead to reduced afterload and increased venous return to the fetal heart respectively [4, 21]. Both pathophysiological mechanisms attributing to the development of cardiomegaly and heart failure. Further fetal fluid imbalance, secondary to developing cardiac dysfunction and/or fetal anaemia can result in increased production of fetal urine [4, 9]. This can be evidenced on ultrasound by the presence of polyhydramnios. In cases of worsening polyhydramnios, maternal discomfort can ensue, and increase the risk of preterm birth, potentially necessitating the requirement for amnioreduction [19, 21]. In conjunction, persistent sequestration and destruction of fetal erythrocytes within the vascular channels of the chorioangioma can precipitate the development of tumour haemorrhage and microangiopathic haemolytic anaemia in the fetus [22, 23]. Further extracorporeal pooling of fetal blood within the intravascular spaces of the chorioangioma can also contribute to the development of fetal anaemia [21]. Such changes can be detected prenatal by elevated MCA PSV indices (>1.5 MoM) on ultrasound, as seen in our case [19]. Over time, persistent shunting and worsening anaemia can result in the evolution of fetal hydrops, whereby the risk of fetal demise increases dramatically [5].

Many large chorioangiomas can be managed supportively, aiming to rectify the pathophysiological complications without directly targeting the tumour itself. Management options would be tailored to the complications that arise, but can include IUT for fetal anaemia; amnioreduction, sulindac or indomethacin for polyhydramnios and digoxin for fetal cardiac dysfunction [6, 9, 24, 25]. However, some of these interventions are not without risk, such as cord thrombosis and haemorrhage with an IUT [26].

When the condition of the fetus continues to decline despite implementing supportive measures, and the development of fetal hydrops ensues (as with our case), the decision for definitive therapy to arrest the placental tumour circulation may be warranted [12]. Devascularisation techniques reported within the literature include laser ablation, bipolar coagulation, radiofrequency ablation and surgical clip application, with fetoscopic laser ablation being the most frequent intervention reported [25, 27, 28]. Importantly, tumour size, vascularity and placental position combined with its association to the umbilical cord insertion can also be factors that influence the choice of surgical intervention to use. In cases of large diameter arterio-venous vessels that are deep-feeding within the tumour, laser ablation may be an inappropriate choice due to the high risk of vessel rupture and haemorrhage [12, 21, 27]. Furthermore, for anteriorly positioned placentas, the ability to insert the equipment safety and ensure adequate visualisation of the tumour may prove too difficult to perform laser ablation [12]. The ability to safely grasp the main feeder vessel and perform bipolar coagulation can also be hindered when in close association with the umbilical cord insertion, again risking vessel rupture [19]. As such, RFA can be a potential therapeutic option for such cases, as noted within our case history which demonstrated a large deep central feeder artery in close proximity to the cord.

As an *in utero* modality, radiofrequency ablation (RFA) is still within its infancy for the treatment of placental tumours. RFA employs targeted high-energy electrical frequencies, approximately 500 kHz, that generates heat within the tissue to destroy the tumour cells. After a prolonged period of sustained tissue temperature (50–100 °C), tissue coagulation and permeant degradation of cells occur [12, 28, 29]. Peak temperatures are limited to ensure tissue boiling, vaporisation and carbonisation does not occur [12].

Currently there are five previously reported cases of RFA for placental chorioangiomas, with variation in neonatal outcomes (Table 1). Agarwal et al. and Yulia et al. both report successful pregnancy outcomes with the use of RFA, whereas Lim et al., Saeed et al. and Zhao et al. all disclose adverse outcomes for the fetus either on the day or following day of the operation [8, 12, 19], [20], [21]. All cases reported at least a one prenatal fetal complication including fetal anaemia, polyhydramnios, cardiomegaly and/or fetal hydrops, whereby interventional therapy was warranted. Two cases (Lim et al. and Zhao et al.) reported the development of fetal hydrops secondary to placental chorioangioma, similar to the presentation of the fetus in our case report. However, both cases sadly resulted in fetal demise either intra or postoperatively. For the two cases that resulted in a successful interventional outcome, the fetuses either had anaemia or mild cardiac abnormalities. In our case, the fetus had several complications involving multiple organ systems. Overall, these successful cases likely carried less risk of fetal mortality when compared to our case report. To the best of our knowledge this case is the first recorded instance of a successful postnatal outcome following RFA for a large placental chorioangioma, whereby the fetus was complicated by fetal hydrops.

**Table 1:** Recorded cases of placental chorioangiomas treated with radiofrequency ablation.

Author	Year	Age of patient, years	Gestation at diagnosis	Size of placental chorioangioma, mm	Fetal complications	Intrauterine therapy	Gestation at delivery	Postnatal outcome
Agarwal [8]	2023	24	–	76×57×107	Fetal anaemia	IUT followed by RFA performed at 25 w & 1 d	37 w+5 d	Live birth
Lim [19]	2015	–	24.43 w	97×86×77	Pulsatile UV, absent A wave in the DV, polyhydramnios, high cardiac output state, fetal hydrops	Bipolar coagulation followed by RFA at 24 w	24.86 w	IUFD
Saeed [20]	2023	38	29 w+6 d	Two masses measuring 100×60×40 and 50×40×20	Fetal anaemia	IUT followed by RFA at 30 w+1 d	30 w+1 d	Stillbirth
Yulia [12]	2019	28	24 w	72×65×38	Mild cardiomegaly and mild pericardial effusion	RFA at 24 w	37 w	Live birth
Zhao [21]	2022	24	24 w	148×99×70 Increased to 170×106×123	Heart failure, fetal anaemia, fetal hydrops	IUT at 27 w followed by RFA 3 days later	27 w	IUFD

IUT, intrauterine transfusion; RFA, radiofrequency ablation; IUFD intrauterine fetal demise; DV, ductus venosus; UV, umbilical vein; EMCS, emergency caesarean section; w, weeks; d, days; mm, millimetres; RFM, reduced fetal movements.

Whilst we demonstrate a positive neonatal outcome overall, the fetus did sustain a period of bradycardia, leading to asystole intraoperatively, which necessitated the need for an emergency caesarean section. Performing the fetal intervention in theatre with the relevant members of the multidisciplinary team present and prepared, allowed for operative complications to be managed promptly. The quick intervention of the fetal medicine team, anaesthetists and theatre staff, alongside the prompt resuscitation performed by the neonatal team aided in the successful outcome for the baby and subsequent postnatal discharge to the community. Additionally, correcting the fetal anaemia just prior to delivery likely contributed to a better neonatal outcome. Potentially serial IUTs alone may have been a sufficient management plan to prolong the gestational age of this fetus. However, one would need to balance this decision between the suspected latency period between each IUT, leading to recurrent fetal anaemia, and the additional fetal complications detected. A short latency between each IUT may warrant definitive intrauterine treatment, despite the high risks associated, particularly if the fetus has hydrops. Importantly, intrauterine intervention should be considered when the fetus is of a viable gestational age, given the potential for expedited delivery to occur.

This case and prior literature clearly highlights the hazardous nature of prenatal interventional therapy for placental chorioangioma. The management and treatment of placental chorioangiomas still remains a significant challenge for fetal medicine specialists.

## Conclusions

Large placental chorioangiomas are a rare occurrence, however, when associated with fetal complications present a high incidence of adverse perinatal outcomes. Early prenatal diagnosis, close antenatal surveillance and timely intervention are key measures to improve perinatal outcomes. Ultrasound and colour flow Doppler remain the key investigations for diagnosis and monitoring, allowing clinicians to detect any early signs of fetal compromise and consider the most appropriate fetal interventions in a timely way. Individual approach is required in such cases. Importantly, tumour size, position, location of major vascular networks and associated fetal morbidity can help determine the appropriateness and choice of prenatal intervention required. *In utero* interventions require thorough and precise planning, surgical expertise and a multidisciplinary team effort to ensure improved fetal and neonatal outcomes. To the best of our knowledge this is the first recorded case of a successful postnatal outcome following RFA for a large placental chorioangioma, whereby the fetus was complicated by fetal hydrops. However, additional research is required to explore the appropriate indication and optimum choice of intrauterine interventions for managing large chorioangiomas.
